# Understanding individual resilience in the workplace: the international collaboration of workforce resilience model

**DOI:** 10.3389/fpsyg.2015.00073

**Published:** 2015-02-04

**Authors:** Clare S. Rees, Lauren J. Breen, Lynette Cusack, Desley Hegney

**Affiliations:** ^1^School of Psychology and Speech Pathology, Faculty of Health Sciences, Curtin University, Perth, WA, Australia; ^2^School of Nursing, The University of Adelaide, Adelaide, SA, Australia; ^3^School of Nursing and Midwifery, The University of Southern Queensland, QLD, Australia

**Keywords:** resilience, workplace, health professionals, burnout, professional, theoretical model, stress disorders, post-traumatic

## Abstract

When not managed effectively, high levels of workplace stress can lead to several negative personal and performance outcomes. Some professional groups work in highly stressful settings and are therefore particularly at risk of conditions such as anxiety, depression, secondary traumatic stress, and burnout. However, some individuals are less affected by workplace stress and the associated negative outcomes. Such individuals have been described as “resilient.” A number of studies have found relationships between levels of individual resilience and specific negative outcomes such as burnout and compassion fatigue. However, because psychological resilience is a multi-dimensional construct it is necessary to more clearly delineate it from other related and overlapping constructs. The creation of a testable theoretical model of individual workforce resilience, which includes both stable traits (e.g., neuroticism) as well as more malleable intrapersonal factors (e.g., coping style), enables information to be derived that can eventually inform interventions aimed at enhancing individual resilience in the workplace. The purpose of this paper is to introduce a new theoretical model of individual workforce resilience that includes several intrapersonal constructs known to be central in the appraisal of and response to stressors and that also overlap with the construct of psychological resilience. We propose a model in which psychological resilience is hypothesized to mediate the relationship between neuroticism, mindfulness, self-efficacy, coping, and psychological adjustment.

## INTRODUCTION

Occupational stress is a universal phenomenon that is associated with several deleterious consequences such as negative physical and mental health outcomes (Kakiashvili et al., 2013) and a number of negative organizational outcomes such as impaired work performance and high turnover (Bridger et al., 2013). Health professionals are one occupational group who appear to be particularly vulnerable to the experience of high levels of workplace stress (Santos et al., 2010). The experience of occupational stress has been consistently linked to negative individual outcomes such as high rates of depression and anxiety, burnout, secondary traumatic stress, and compassion fatigue (Figley, 2002; Bride et al., 2007) as well as a number of negative patient and organizational outcomes such as reduced work performance and comprised patient care (Tan et al., 2014). Understanding the factors that impact upon employee workplace stress is essential in the subsequent development of initiatives that may positively impact upon levels of stress and thus reduce the associated negative outcomes.

Some research has focused on context-specific occupational stress, such as for professionals working in “high-death” contexts such as cancer support and palliative care. Systematic reviews have highlighted a high prevalence of burnout in professionals working in cancer services (Trufelli et al., 2008; Pereira et al., 2011) and in-depth qualitative interviews highlighted the emotional demands of working in cancer and palliative care services and the need for the inculcation of self-care into practice (Breen et al., 2014). Another focus of the literature has been role-specific such as the occupational stress experienced by nurses (Drury et al., 2014; Hegney et al., 2014), oncologists (Granek et al., 2012a,b) psychologists (D’Souza et al., 2011), and school counselors (Butler and Constantine, 2005).

It is important to clarify some of the constructs that are often used interchangeably within the occupational stress literature. As a starting point the term “stress” needs to be clarified. It is important to note that stress responses exist on a continuum from mild and short-lived experiences through to more severe and enduring stress. Occupational stress occurs primarily in response to how an individual appraises an occupational stressor (Ashkanasy et al., 2004) and there are individual differences in how people engage in this process of appraisal (Finlay-Jones, 2014). High work-related stress can lead to impaired work performance, potentially compromising client care (Barnett et al., 2007) and chronic stress may impair attention (Skosnik et al., 2000) and decision-making skills (Starcke and Brand, 2012). The term “psychological distress” refers to negative psychological consequences such as symptoms of stress, anxiety, and depression (Finlay-Jones, 2014).

The experience of enduring and high levels of stress can increase the likelihood of a person developing burnout, a syndrome first introduced by Pines and Maslach (1978) and consisting of emotional exhaustion, depersonalization and a sense of low personal accomplishment. Burnout is associated with symptoms of cognitive impairment, such as memory loss, concentration difficulties, and problems solving complex tasks. Burnout has also been associated with depersonalization and an inability to work effectively (Stamm, 2010), increased absenteeism, reduced productivity, and negative effects on the ability of the individual to deliver safe care, compromising patient safety (Mealer et al., 2012).

A related but slightly different construct is compassion fatigue, a type of occupational burnout that has been found to be particularly associated with caregiver stress and thought to occur as a result of providing ongoing empathy and compassion to others but neglect of one’s own self-care (Figley, 1995). Similarly, vicarious traumatization and secondary traumatic stress are considered to be conditions that are brought on by working in settings where exposure to traumatic events or situations is common (Canfield, 2005). On the flip side, researchers have also been interested in studying the positive outcomes that may be associated with occupational stress. For example, some individuals may find stressors motivating and the experience may elicit feelings of personal satisfaction and accomplishment (Finlay-Jones, 2014). Given the clear relationship between the experience of occupational stress and various negative individual and organizational outcomes, researchers have become increasingly interested in exploring factors that might serve to either exacerbate or mitigate the influence of stress on employees. One such construct that has gained considerable research interest is that of psychological resilience.

Psychological resilience has been defined as the ability of a person to recover, re-bound, bounce-back, adjust or even thrive following misfortune, change or adversity (Garcia-Dia et al., 2013) and is widely acknowledged to be a complex, dynamic and multi-dimensional phenomenon (Waugh and Koster, 2014). Studies have now shown a link between psychological resilience and various mental health outcomes such as burnout, secondary traumatic stress, depression, and anxiety (Mak et al., 2011; Mealer et al., 2012; McGarry et al., 2013; Lu et al., 2014). For example, a study by Mealer et al. (2012) included 744 intensive care nurses working in the United States and found that high resilience was associated with a lower prevalence of burnout, symptoms of anxiety and depression and symptoms of post-traumatic stress disorder. Similarly, a study by McGarry et al. (2013) conducted in Australia with health professionals working in a pediatric hospital, found that high resilience was associated with lower prevalence of burnout, symptoms of anxiety and depression and symptoms of post-traumatic stress disorder and low psychological resilience was associated with higher secondary traumatic stress.

As described, studies with various occupational groups have found that an individual’s level of psychological resilience is significantly related to mental health outcomes. Individuals who score more highly on measures of individual resilience also score more highly on measures of psychological well-functioning and *vice versa*. Whilst this relationship is clear, what is less clear from the extant literature is how individual resilience exerts its impact on mental health outcomes. What is the relative importance of psychological resilience in determining outcomes when considered alongside other salient individual psychological factors (e.g., self-efficacy)? The aim of developing the current model is to advance understanding about the role of individual psychological resilience and its impact on psychological adjustment. Whilst, numerous studies have found relationships between psychological resilience and various psychological outcomes, little is known about how other individual psychological variables influence this relationship. Therefore, the aim of developing the present model is to study resilience and psychological adjustment from a more inclusive perspective, where other key psychological variables can be accounted for simultaneously when attempting to understand the relationship between psychological resilience and psychological adjustment. Specifically, in this paper we propose that psychological resilience mediates the relationship between several key individual psychological variables and general psychological adjustment.

## THEORETICAL BACKGROUND

### PSYCHOLOGICAL RESILIENCE

Interest in the concept of psychological resilience has burgeoned in the last decade with researchers across various discipline areas (e.g., psychology, nursing, business) investigating the relationship between an individual’s level of psychological resilience and various outcomes ranging from reported levels of stress, burnout, compassion fatigue, and general indicators of well-being (Garcia-Dia et al., 2013). Although a number of authors have proposed that psychological resilience is a dynamic phenomena (Waugh and Koster, 2014) and is influenced by many inter and intrapersonal factors as well as environmental factors, another school of thought regards psychological resilience as a more stable and enduring personality trait that impacts upon an individuals self-regulatory processes (Block and Block, 1980).

Despite some agreement as to the definition of psychological resilience and clear findings that it is related strongly to a number of important individual outcomes, it is surprising that there is no leading, unified theoretical model of individual workforce resilience that can be applied across disciplines and organizational settings. Determining the relative importance of psychological resilience in explaining mental health outcomes when other key psychological variables are also examined, is a key step in the development of interventions to improve the psychological adjustment of employees working in high stress settings. Often interventions tend to adopt an over-inclusive approach, whereby several different strategies and techniques are included in the one intervention in the hope that something will be effective. We suggest that the most powerful interventions will need to be drawn from theory and that the necessary groundwork needs to occur before meaningful interventions can be devised.

Windle et al. (2011) reviewed over 270 published research articles and evaluated the psychometric properties of several published resilience measures. They noted that the majority of the measures were not developed on the basis of a clear theoretical model of psychological resilience. The authors conclude that psychological resilience is currently measured from a multi-level perspective and measures include a variety of components thought to constitute psychological resilience. Such components include: optimism, self-esteem, personal competence, social competence, problem-solving skills, self-efficacy, social resources, insight, independence, creativity, humor, control, hardiness, family cohesion, spiritual influences, and initiative (Windle et al., 2011). Clearly, a number of different components have been proposed to be a part of the overall construct of psychological resilience. Disentangling the relationship between psychological resilience and related psychological variables and mental health outcomes can be achieved by creating a testable model.

### THE BIOPSYCHOSOCIAL MODEL

In developing a testable, theory-driven model of individual workforce resilience we have synthesized key psychological models as well as empirical results from previous studies to distil what we consider to be the key psychological variables related to individual workforce resilience. The psychological models reviewed fall within an overarching biopsychosocial model of emotional functioning (Melchert, 2011). This general model presumes that an individual’s emotional health is determined by a convergence of several factors. First, biology exerts a strong influence upon an individual’s vulnerability to adverse mental health outcomes. Individuals may inherit a generalized biological vulnerability to emotional problems in the form of heightened emotional reactivity, or a more specific genetic predisposition to certain mental health problems such as bipolar depression or anxiety. The psychosocial element of the model proposes that biological predispositions then interact with an individual’s particular set of environmental and social circumstances. For example, the type of parenting one receives, exposure to traumatic life events, and socio-cultural factors combine to determine an individual’s overall vulnerability to emotional disorder.

The biopsychosocial model is an ideal framework with which to consider psychological resilience because it rests on the notion that clinical problems have multiple interacting causes and contributing factors. We now turn to some specific psychological constructs of particular relevance to understanding individual resilience. Each of the constructs aligns with different aspects of the biopsychosocial model. For example, neuroticism represents an important biological vulnerability to emotional difficulties, whereas mindfulness, self-efficacy, and coping are considered psychosocial factors. It should be noted that whilst these constructs are conceptually distinct, they cannot be presumed to be completely independent of one another. As previously explained, the purpose of developing the current model is to begin to account for areas of overlap in psychological constructs related to psychological resilience and psychological adjustment. Each of these constructs will now be discussed in detail.

### NEUROTICISM

The study of normal emotional experience has shown that being “highly strung” or “emotional” is strongly genetically determined (Eaves and Eysenck, 1976) and related to a factor interchangeably referred to as neuroticism, trait anxiety, negative affect or trait negative affect. Neuroticism refers to the tendency to experience enduring negative emotional states such as anxiety, guilt, anger and depression more frequently, intensely, and readily, and for a more enduring period of time. This dimension of personality is considered an important biological vulnerability to the development of emotional disorders in general. Similarly, Spielberger (1972) developed a state-trait process model of anxiety in which trait anxiety is considered a personality trait and different to the more transient state-anxiety. Finally, Clark and Watson (1991) developed a Tripartite Model of affect in which they concluded that anxiety and depression share an underlying common component characterized by generalized distress that they termed “negative affect.” Negative Affect refers to the experience of non-specific distress or unpleasant emotionality. Each of these related constructs have consistently been shown to be reliably associated with the development of emotional disorders (Barlow, 2002).

Empirical studies have consistently found a relationship between high levels of Neuroticism or Negative Affect and negative mental health outcomes such as symptoms of anxiety, depression, and psychological disorders (Rees et al., 2014). A recent study exploring the impact of this personality variable upon the mental health outcomes of employed nurses found significant relationships between trait negative affect and scores on depression, anxiety, stress, secondary traumatic stress, and burnout (Drury et al., 2014). The significant influence of TNA or neuroticism upon negative health outcomes has also been confirmed in other studies (Cañadas-De la Fuente et al., 2015).

A number of studies have found that higher levels of neuroticism are associated with lower levels of individual psychological resilience (Bakker et al., 2006; Campbell-Sills et al., 2006; Lu et al., 2014). A study by Lu et al. (2014) found that psychological resilience was negatively correlated with neuroticism and that resilience mediated the relationship between neuroticism and negative affect.

### MINDFULNESS

Dispositional mindfulness refers to a trait-like tendency to experience and express mindful qualities (e.g., non-judgment) and behavioral qualities (e.g., acting with awareness rather than automaticity). Low mindfulness is characterized by an inability to attain a de-centered perspective on events and a tendency to respond reactively and inflexibly to negative thoughts and emotions (Teasdale, 1999). The benefits of cultivating a mindful state have been recognized for some time in organizational settings where it has been referred to as “collective mindfulness” (Weick et al., 1999). A number of studies have found an association between the inability to detach from experience (low mindfulness) and symptom severity of anxiety and mood disorders (Fennell, 2004; Roemer et al., 2009; Arch and Craske, 2010). Garland (2007) and Garland et al. (2011) has posited that a mindful state may facilitate disengagement from an initial appraisal of a stressor into a metacognitive state whereby thoughts about the stressor are appraised with greater perspective and less habitual, emotion-laden responses. In this way, the individual is able to de-center from an experience in a way that enables a more balanced appraisal of events to occur. A recent study by Viladarga et al. (2011) investigated the relationship between level of mindfulness, workplace variables (e.g., workload, co-worker support) and burnout among a sample of 699 addiction counselors in the United States. They found that mindfulness was the strongest predictor of burnout, over and above the variance explained by workplace factors. Increasingly, studies are proposing that mindfulness is an important characteristic of a resilient individual. In a recent review of mindfulness and individual resilience to trauma, Thompson et al. (2011) suggest that a mindful and accepting orientation toward experience may promote psychological resilience following trauma.

### SELF-EFFICACY

Self-efficacy is an individual’s belief that he or she can perform a selected task (Bandura, 1977). Bandura proposed that self-efficacy underpins whether an individual will engage an approach to achieve the task and the effort expended in engaging in the approach. Meta-analyses demonstrate a moderate correlation between self-efficacy and workplace performance (Stajkovic and Luthans, 1998; Judge et al., 2007). Employees who report higher levels of perceived self-efficacy have been found to have lower levels of anxiety, better coping skills and lower intentions of leaving their workplace (Saks, 1994). Self-efficacy is highly correlated with psychological resilience (Li and Nishikawa, 2012) with some conceptualizing it as one of the major components (Rutter, 1987) and referring to a resilient self-efficacy. Indeed, self-efficacy is closely related to the concept of personal competence that makes up one of the five sub-scales of the Connor-Davidson Resilience Scale (CD-RISC).

### COPING

Coping is a process of adjustment following an adverse event. Typically, coping strategies are categorized as either problem-focused, aimed at addressing the practicalities of a situation, or emotion-focused, aimed at reducing the psychological and emotional impact of a stressor (Lazarus and Folkman, 1984). A study of 518 nurses working in Australia and New Zealand showed that their use of problem-focused coping strategies was associated with better mental health while the use of emotion-focused strategies was associated with reduced mental health (Chang et al., 2007). Others have posited coping to comprise multi-dimensional strategies that may be active/adaptive, such as planning, positive reframing of stressors, and engaging professional help, or passive/maladaptive, such as venting, substance use, and disengagement (Carver et al., 1989). In the workplace, positive reframing and support seeking coping is associated with greater job satisfaction and the use of avoidant coping with less job satisfaction (Welbourne et al., 2007) and emotional support is associated with workplace absenteeism (Karlsson et al., 2010). Use of active coping has been found to be positively associated with psychological resilience and a mediator of the relationship between self-efficacy and individual resilience (Li and Nishikawa, 2012).

## A MODEL OF INDIVIDUAL WORKFORCE RESILIENCE

**FIGURE 1:**
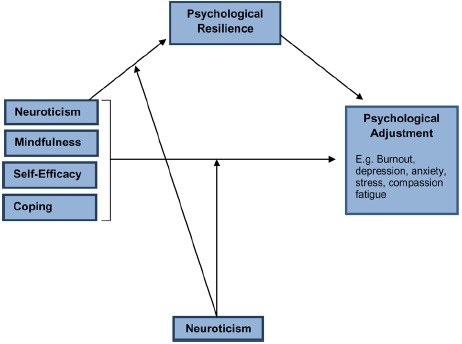
**The ICWR-1 model of individual workforce resilience**.

As reviewed, psychological resilience is a centrally important construct in understanding how individuals respond to workplace stressors and appears to be a major determinant of whether or not certain unfavorable outcomes such as burnout, compassion fatigue, anxiety, or depression ensue. However, as has already been discussed, psychological resilience is a multi-dimensional construct and its relationship to other variables such as neuroticism, mindfulness, self-efficacy and coping in the context of workplace stress is not clear. Figure 1 displays the proposed model of individual workforce resilience. This model is primarily concerned with the intrapersonal factors that converge to explain individual psychological adjustment. An illustrative example of how the components of the model interact will now be provided.

The starting point for the model is the basic proposition that an individual will at some point be exposed to workplace stressors (either acute or chronic). As already reviewed, *Neuroticism* exerts a significant influence on psychological well-being and it is known from numerous studies that it is strongly related to negative outcomes such as stress and burnout (Rees et al., 2014). Individuals high on Neuroticism have a tendency to be more emotionally reactive in general and our own research with the nursing workforce has confirmed Neuroticism (trait negative affect) to be the strongest predictor of stress, anxiety and depression after controlling for other workplace variables such as length of employee experience and age (Hegney et al., 2014). Studies have also shown that Neuroticism is negatively correlated with psychological resilience. As neuroticism is a stable characteristic that has a broad influence upon general psychological functioning it is included in the current model as a moderator variable.

The next component of the model is *mindfulness*. Individuals have varying degrees of psychological awareness or the ability to be mindful. If a person is low on mindfulness it means that when faced with a stressor they will be less likely to be able to detach from what is happening and get distance from the situation, or be able to reflect on what is happening. They are more likely to become immersed in the situation and overwhelmed emotionally by it. Alternatively, those who are mindful can mentally step back and think about what is going on and what can be done about it.

The next component of the model is *self-efficacy*; whether or not a person believes they can change a situation or do something to cope with it. Self-efficacy is multi-determined in that it will be impacted by a person’s past experiences, their spiritual beliefs, their core beliefs and so forth. The key reason for including self-efficacy in the model is that it logically impacts on the way a person will attempt to manage a stressor and is thus intrinsically linked to the next part of the model; *coping*. For example, if a person does not believe that they can do anything to alter a stressful situation (low-self-efficacy) they will be more likely to engage in passive coping such as avoidance and substance use. Alternatively, if a person believes that there is something they can do about a current stressor they will be more likely to engage in effective active coping strategies, such as seeking social support, problem-solving, and the use of cognitive-reappraisal. This type of coping is associated with better outcomes in the face of a stressor. The final part of the model is *Psychological Adjustment* and represents the main outcomes or dependent variables in the model. Psychological adjustment may be determined by measuring symptoms of stress, depression, anxiety, burnout, and compassion fatigue.

In order to test the proposed model we offer the following hypotheses. First, we predict a significant negative relationship between neuroticism and psychological adjustment; a significant positive relationship between mindfulness and psychological adjustment, self-efficacy and psychological adjustment and coping and psychological adjustment. The central hypothesis that underpins the model is that each of these direct relationships will be mediated by psychological resilience. Furthermore, it is hypothesized that neuroticism will act as a key moderator variable, influencing the mediational properties of resilience upon psychological adjustment for each of the predictor variables (mindfulness, self-efficacy, coping).

As resilience is the central construct of interest in the model some options as to how to best measure the construct will now be considered. We propose that the construct of psychological resilience is best captured by a measure that includes both aspects of trait-resilience as well as the more dynamic and the more permutable aspects of psychological resilience. In a recent review of 15 different self-report measures of resilience, Windle et al. (2011) identified three measures (CD-RISC; Resilience Scale for Adults, RSA; Brief Resilience Scale) that scored the most highly in terms of psychometric qualities such as internal consistency and construct validity. The CD-RISC is the most widely used measure of resilience and has been translated into several languages and validated in many different countries including China, South Africa, Iran, USA, Australia, and Brazil. It consists of five factors (personal competence, acceptance of change and secure relationships, control, spiritual influences). The scale was developed to capture a blend of trait-aspects of resilience (such as hardiness) as well as other aspects of resilience such as self-confidence, possessing social problem solving skills and the role of faith and spirituality (Connor and Davidson, 2003). We suggest that the CD-RISC represents a sound measure of resilience that is suitable for large-scale surveys not only due to its good psychometric properties but also because it is a relatively brief measure.

Whilst the proposed model does not claim to capture every salient aspect of determining individual workforce resilience, we believe it represents an initial testable model that includes the “big-players” in terms of predicting psychological adjustment and thus understanding the role of resilience in this relationship. It should be acknowledged that this preliminary model may not account for the true complexity of the relationship between each of the included variables. Whilst we have included neuroticism as the key moderator in the model it is possible that some of the other variables will also have a moderating effect on resilience and psychological adjustment. We regard this model as an important starting point and believe it will further evolve following initial testing, with new pathways being proposed as data is gathered and analyzed. It is possible that some of the hypothesized relationships represented in the model will not be supported. After rigorous testing utilizing the best available measures, it may be the case that some variables are dropped from the model with new ones added. However, inclusion of new variables should also be justified theoretically. We also suggest that initial tests of the model are carried out with large samples, across different occupational groups.

## CONCLUSION

Workplace stress has serious implications for the quality of an employee’s work and their general psychological functioning. Research investigating the relationship between psychological resilience and workforce outcomes has consistently shown psychological resilience to be strongly related to levels of psychological distress. However to date, the research lacks a model of individual workforce resilience that enables direct testing of the relationship between similar constructs such as self-efficacy that overlap with psychological resilience. The central premise of this model is that previously observed relationships between variables reported in the literature, such as coping and psychological adjustment, will be explained by resilience. If psychological resilience is found to mediate the relationships among variables, it will provide important evidence for specifically targeting individual resilience in an effort to promote healthy psychological adjustment for employees in high stress work settings. It is very clear that there is a need for such interventions; particularly for certain occupational groups such as those working in palliative care settings where rates of compassion fatigue and burnout are high.

We are currently testing this model as part of our International Collaboration on Workforce Resilience - 1 (ICWR-1). This international collaboration of researchers is currently involved in collecting data from large numbers of nurses working in various settings in Australia, Singapore, Hong Kong, and Canada. Our initial testing of this model will take place with the nursing workforce but we expect to extend this testing to other occupational groups in the near future.

### Conflict of Interest Statement

The authors declare that the research was conducted in the absence of any commercial or financial relationships that could be construed as a potential conflict of interest.
